# Current Status and Policy Planning for Promoting Age-Friendly Cities in Taitung County: Dialogue Between Older Adults and Service Providers

**DOI:** 10.3390/ijerph15102314

**Published:** 2018-10-21

**Authors:** Li-Chuan Liu, Hsien-Wen Kuo, Chiu-Chu Lin

**Affiliations:** 1 Department of Public and Cultural Affairs, National Taitung University, Taitung 95092, Taiwan; 2 Institute of Environmental and Occupational Health Sciences, National Yang-Ming University, Taipei 11221, Taiwan; hwkuo1106@gmail.com; 3 Health Promotion Section, Public Health Bureau, Taitung County, Taitung 95043, Taiwan; jill480730@yahoo.com.tw

**Keywords:** age-friendly city (AFC), Taitung County, older adults (demanders), service providers

## Abstract

The World Health Organization has promoted age-friendly city (AFC) projects in response to the aging population. Taiwan has also promoted AFC policies. This study was conducted in Taitung County, where 15.37% of the population is older adults in Taiwan. The aim was to understand the perceptions of older adults and service providers with regard to the current status of AFC policies to influence future policies. The participants of this study were older adults and service providers in various regions of Taitung. Quantitative questionnaires were completed by older adults and qualitative interviews were held with focus groups. The older adults were the most satisfied with the AFC domains of “respect and social inclusion” and “community and health services”, and the least satisfied with “transportation” and “civic participation and employment”. Homogeneity existed between the older adults’ satisfaction levels in different regions and the service providers’ opinions; however, there were notable differences between them. Both economic development and the ethnicity of groups in different regions are influential factors that determine the success of government policies. In promoting AFC policies, local governments should consider their applicability based on local conditions and resources to meet the needs of the aging population in rural areas.

## 1. Introduction

Population aging has been observed in countries around the world. Older adults are vital resources for their families, communities, and the economy. Therefore, most countries are committed to establish policies to build age-friendly environments for older adults that enable them to exercise their abilities and values and promote their health, well-being, and quality of life. The World Health Organization (WHO) believes that establishing and maintaining an age-friendly environment should be the core of urbanization policies with regard to an aging population, which promotes the concept of “aging in place” by pursuing active and healthy aging [1,2,3].

To establish an age-friendly environment, the WHO launched the “Global Age-Friendly Cities Project” in 2005, of which, Canada was the first to implement the project. The WHO’s “Global age-friendly cities: a guide” was published in 2007. A major goal of this guide is to improve older adults’ quality of life through promoting their health, participation, and safety. In addition, the core value of active aging is promoted, which is to enable older adults to continue to live in their home and their communities to achieve the goal of aging in place [2].

The concept of an age-friendly city (AFC) comprises eight domains: outdoor spaces and buildings, transportation, housing, social participation, respect and social inclusion, civic participation and employment, communication and information, and community and health services. The AFC concept promotes continuous participation of older adults in social, cultural, spiritual, economic, and civil affairs rather than participation in only the labor market or general physical activities. Governments should also promote and implement action plans for active aging by offering policies and programs that improve social and natural environments [1]. Based on this concept, the WHO’s Global Network for Age-Friendly Cities and Communities was established in June 2011. Many cities have subsequently developed and promoted AFC projects.

In response to this initiative, the Ministry of Health and Welfare launched an AFC demonstration project in Chiayi City, Taiwan in 2010. AFC was designed to change the public’s attitude toward older adults. On the basis of the eight domains of an AFC developed by the WHO, the Chiayi City Government actively encouraged the participation of older adults in social activities to create a livable and friendly environment for different generations. Subsequently, eight other counties and cities in Taiwan joined the AFC project in 2011, namely Taipei City, New Taipei City, Taoyuan County, Hsinchu City, Nantou County, Tainan City, Kaohsiung City, and Taitung County. By 2013, the mayors of all counties and cities in Taiwan promised to promote the AFC project. Taiwan is the first country in Asia to use an AFC project as a national flagship project [4].

“Aging in place” for older adults is a complex issue. Apart from supporting older adults in their communities and offering basic economic and social support, additional effort is required to provide supportive services to eliminate limitations and obstacles for older adults regarding social participation [5,6]. Although the AFC project has become a pertinent program for the aging population in Taiwan, its development should consider the trend of the aging population as well as the development of infrastructure in counties and cities, thereby requiring a diversified policy approach. Moreover, aging is an ecological model when assuming that physical and social environments affect aging in place and quality of life during the aging process [7]. That is, how an age-friendly social and physical environment is established determines how older adults age in place and maintain a high quality of life [8,9,10].

Taitung County, located in eastern Taiwan, has actively applied the AFC project since 2011 and has adopted the related concepts of the project in each bureau’s core tasks. By integrating resources from different departments, Taitung County has worked toward creating a friendly urban environment to maximize the health of older adults. During the implementation of the project, an AFC Promotion Committee was established and integrated into the former Healthy City Committee. Researchers and other experts with regional and industry sector knowledge formed a consultancy team and developed planning policies based on the eight domains of an AFC. However, Taitung County has been called “back mountain” for a long time, which means that the County falls behind in the construction of every respect. There are seven indigenous groups, including the Ami tribe, the Pinuymayan tribe, the Bunun tribe, the Yami tribe, the Rukai tribe, the Paiwan tribe, and the Kavalan tribe, and the immigrants at later period have contributed to the diverse humanity of current Taitung. The Aboriginal people have been disadvantaged by economic disadvantage, resource alienation, and political oppression. Because of a lack of public transportation and medical resources and because of the influence of pervasive cultural and living habits, the county’s mortality rate is the highest among the cities and counties in Taiwan. Until September 2017, older adults in Taitung County accounted for 15.37% of the county’s population [11]. According to the Ministry of the Interior, the average life expectancy of the inhabitants of Taitung County in 2016 was 75.5 years, which was approximately five years shorter than the average life expectancy in Taiwan [12]. Therefore, it is crucial that Taitung County develops appropriate AFC policies to meet the needs of its growing population because it is remote and exhibits a large older adult population. Taitung County’s core goals are to increase average life expectancy and to enhance the health of its older adults.

Therefore, this study explored the following issues. (1) Perceptions of older adults (demanders) regarding the current age-friendly environment, (2) service providers’ opinions of age-friendly needs, and (3) dialogue analysis between the older adults (demanders) and service providers. By using a dialogue analysis, this study aimed to establish the direction of the AFC project by assisting Taitung County with the development of its AFC policies.

## 2. Materials and Methods

### 2.1. Research Framework and Methods

**Figure 1:**
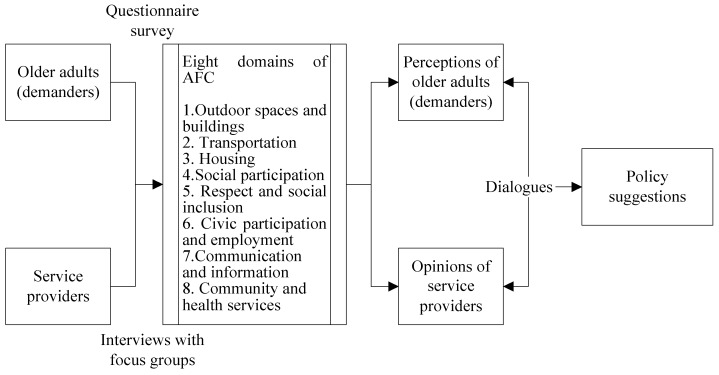
Research framework.

Because planning and establishing an AFC involves multiple stakeholders and requires an understanding of the different geographical environments and resources between urban and rural areas within Taitung County, it is challenging to conduct an AFC analysis and to develop effective policies. Therefore, to understand the needs of different stakeholders, this study employed a triangulation method by adopting two research methods to develop a policy that accounts for the various issues. These two methods comprised a quantitative questionnaire survey and qualitative interviews with focus groups. This study was conducted using these two methods on different participants on the same topic. The research framework is shown in Figure 1.

The quantitative questionnaire survey was used for describing, interpreting, and exploring the various issues concerning older adults and relevant service providers; the characteristics of the parent population were deduced according to the results of the sample data [13]. This study, which was based on AFC’s eight domains, examined the perceptions older adults had of those domains, the findings of which were used to develop suggestions for governments wanting to promote AFC projects in the future. The qualitative data were acquired from the focus groups. The results express the experience and opinions of various stakeholders [14,15]. Because focus groups are often used to assist decision-making, this method has also been applied for exploring sensitive health issues [16,17,18]. Focus groups were used to obtain both broad and specific opinions concerning policies from service providers in an AFC context.

This study adopted both qualitative and quantitative approaches because this combination includes various methods with which we could address the research questions. Though combined methods research, this study would produce more inclusive, diversity, and complementary results than either method [19,20].

### 2.2. Research Participants and Contents

People aged over 65 years and aborigines (the average life expectancy of aborigines is lower than that of non-aboriginenon-aborigines in Taiwan. Therefore, aborigines aged over 55 years are considered as older adults) over 55 years in all townships within Taitung County were the participants of the questionnaire survey. The quantitative questionnaire survey based on eight domains of AFC’s checklist [2] and adjust question by situation of Taitung County. The total questions are in regard to 64 items, including “Outdoor spaces and buildings” (seven items), “Transportation” (11 items), “Housing” (6 items), “Social participation” (nine items), “Respect and social inclusion” (eight items), “Civic participation and employment” (10 items), “Communication and information” (seven items), and “Community and health services” (six items). Older adults rank satisfaction with respect to the different domains from high to low or best to worst. A five-point scale consisting of “strongly agree, agree, undecided, disagree, and strongly disagree” was used.

This study applied disproportionate stratified sampling (disproportionate stratified sampling was conducted in this study according to the older adult population of each township; 8% in a sample of below 1000 people; 4% in a sample of 1000–10,000 people; and 2% in a sample of over 10,000 people) because of the large differences in the numbers of older adults between the townships to strengthen the representativeness of the samples, which conforms with the distribution of the parent population in Taitung County. The regional sample size and proportional allocation is shown in Table 1.

To understand the different older adult groups in a single township, face-to-face interviews were conducted at places where they gathered, such as communities care centers and tribal culture and health stations in different regions. The questionnaire survey was intended to clarify the needs of the older adults for future AFC project planning.

In addition to the aforementioned older adults, this study considered the unique geography of Taitung County, namely its mountainous region, rift valley, and coastal terrain. Therefore, to clarify the current issues and demands of the older adults in different townships, this study applied regions/townships as variables and incorporated interviews with four focus groups according to the following regions: downtown Taitung (Taitung City and Beinan Township), the South-link region (Taimali Township, Jinfeng Township, Daren Township, and Dawu Township), the East Rift Valley region (Luye Township, Chihshang Township, Guanshan Township, Haiduan Township, and Yanping Township), and the East coast (qualitative focus groups were limited to townships on the mainland of Taitung County, excluding offshore islands such as Lanyu Township and Lüdao Towanship) region (Changbin Township, Chenggong Township, and Donghe Township). Interviews were mainly based on interactions between the participants. Each group was limited to eight to 12 participants [14]. The participants comprised local service providers, government units, and researchers and other experts (Table 2).

During interview process, participants, according to their experience and expertise, were asked to discuss their opinions on the following questions: (1) one or several AFC domains that they were familiar with; (2) the best and worst performing results with regard to the AFC domains and the reasons for such performances; and (3) the current status of each AFC domain and their strengths and weaknesses. The participants’ opinions revealed the current status of the age-friendly policy in Taitung County and the measures to improve it.

### 2.3. Procedures and Data Analysis

The questionnaire surveys were completed from April to October 2015 as face-to-face interviews by visiting community care centers of each township according to the envisaged number of samples. Questionnaire data were recorded and analyzed using SPSS Statistics software. A descriptive statistical analysis of the demographic information was conducted and presented with regard to frequency and percentage to understand the demographics of the samples. Next, items regarding the eight AFC domains were analyzed in terms of frequency, percentage, and mean values to understand the older adults’ opinions of each item.

Four focus groups were held in August 2015 in downtown Taitung, the South-link region, the East Rift Valley region, and the East Coast region. The topics, purposes, methods, and relevant data to be discussed were provided in advance to enable productive group meetings. The focus group interviews were divided into three stages: signing the consent forms, self-introduction, and the discussion. Each interview was recorded and videotaped. The contents were transcribed to ensure intrinsic validity. All focus group interviews were recorded for a total of 10 h and equated to 150,570 words. In addition to ensuring that the qualitative interview data had internal reliability by proofreading the data, we focused more on both internal and external validities. Internal validity ensured the authenticity of the research data, and external validity ensured that the participants’ feelings and experiences were objectively presented [14,15].

Quantitative research was conducted, and a focus group method was applied. Taitung County was divided into four areas. On the basis of the eight AFC domains, the satisfaction of the older adults and the qualitative opinions of the service providers of those areas were compared. The older adults’ perceived feelings directly reflected their views on the eight AFC domains, whereas the service providers’ qualitative opinions revealed their observations and understanding of the older adults’ needs in their service areas. The similarities between their responses regarding the eight AFC domains can be used as a reference for policymakers in Taiwan, while the different views among older adults and service providers is worthy of investigation and in-depth analysis.

## 3. Results

### 3.1. Older Adults: Analysis of Survey Results

Overall, 1090 questionnaires were distributed, 850 of which were collected and 47 were invalid (recovery rate of 73.67%). Cronbach’s α for the overall reliability of the questionnaire was 0.9, revealing that the questionnaire had a high level of internal consistency and reliability (Cronbach’s α greater than 0.7 is considered reliable [21]). The reliability of each domain was above 0.7, revealing that each domain was also internally consistent, as shown in Table 3.

Table 4 presents the demographic information of the interviewees: 61.0% were women and 67.5% were aborigines; 45.6% were aged 65–74 years, 29.0% were aged 75–84 years, and 0.4% were aged above 95 years; 31.8% were illiterate; 31.0% lived with spouses, 23.3% lived with children, and 24.8% lived alone; 50.1% were married and 41.1% were widowed; 15.4% lived in Taitung City, 14.8% in Beinan Township, 10.6% in Chihshang Township, 9.5% in Chenggong Township, and 1.2% in Haiduan Township; 94.3% lived in average households, 2.2% lived in low- and middle-income households, and 3.5% lived in low-income households; 85.1% received allowances and 14.9% did not; over 80% had never volunteered and 2.4% had previously volunteered for more than 8 h a month, revealing that opportunities remain available for older adults to volunteer.

Table 5 shows the older adults’ average scores on the basis of the eight AFC domains in Taitung County. Because of the differences between the number of items of each dimension and the total scores, the average satisfaction levels of the AFC domains were converted to relative scores. The so-called “relative scores” represented the participants’ relative satisfaction with respect to the different domains. By using relative scores, the domains could be compared and analyzed. The equation for relative scores is as follows, (the mean value of each domain ÷ the total score of a specific domain) × 100. The results showed that “Respect and social inclusion” had the highest score (72.0), followed by “community and health services” (68.1), “housing” (67.7), “communication and information” (67.4), “social participation” (65.4), and “outdoor spaces and buildings” (64.8). “Transportation” (50.3) and “civic participation and employment” (9.0) had lower average scores; therefore, improvement is particularly required in those areas.

Further comparison of the average scores of the townships regarding the eight AFC domains (Table 6) revealed that Lüdao Township had the highest score (72.2) and Lanyu Township the lowest score (58.2) with respect to “outdoor spaces and buildings”. Yanping Township had the highest score (76.0) and Jinfeng Township the lowest score (4.5) regarding “transportation”. Jinfeng Township had the highest score (72.7) and Lanyu Township had the lowest score (58.0) regarding “housing”. Yanping Township had the highest score (74.2) for “social participation”, whereas Lüdao Township had the lowest score (50.2). Taimali Township had the highest score (80.4) for “respect and social inclusion” and Lüdao Township had the lowest score (58.8). Daren Township had the highest score of 48.0 and Haiduan Township did not have a score with respect to “civic participation and employment”. Changbin Township had the highest score (73.7) for “communication and information”, whereas Lüdao Township had the lowest score (61.0). Guanshan Township scored the highest (73.6) for “community and health services” and Lanyu Township had the lowest score (57.2).

As for the overall regional analysis, offshore islands extremely lacking in infrastructure and resources had the lowest average scores in the following domains “transportation”, “housing”, “social participation”, “respect and social inclusion”, “communication and information”, and “community and health services”. Excluding offshore islands, the older adults in the East Coast region were the most satisfied (68.4) with “outdoor spaces and buildings”, whereas the older adults in Taitung City were the least satisfied (60.2). The older adults in the East Coast region were the most satisfied (67.0) with “transportation”, whereas the older adults in Taitung City were the least satisfied (35.0). The older adults in the East Rift Valley region were the most satisfied (70.9) with “housing”, whereas the residents in the East Coast region were the least satisfied (66.3). The older adults in the East Rift Valley region were the most satisfied (68.9) with “social participation” and older adults in the downtown area were the least satisfied (61.5). The older adults in the East Coast region were the most satisfied (75.0) with “respect and social inclusion”, whereas the older adults in the South-link region were the least satisfied (67.9). The older adults in the South-link region were the most satisfied (15.1) with “civic participation and employment”, whereas the older adults in the East Coast region were the least satisfied (2.9). The older adults in the East Coast were the most satisfied (69.1) with “communication and information”, whereas the older adults in the South-link region were the least satisfied (65.9). Residents in the East Rift Valley region were the most satisfied (70.6) with “community and health services”, whereas residents in the South-link region were the least satisfied (66.4).

According to the scores of the eight AFC domains, “transportation” and “civic participation and employment” require the most improvement, followed by “outdoor spaces and buildings”. Conversely, significant differences existed between the townships regarding the eight AFC domains. For instance, offshore islands such as Lüdao Township (“social participation”, “respect and social inclusion”, and “communication and information”) and Lanyu Township (“outdoor spaces and buildings”, “housing”, and “community and health services”) had the lowest average scores, revealing that these two townships are remote and are lacking in resources compared with the other townships. However, in all the domains, Taitung City had the lowest score for “transportation” compared with the other townships, revealing that the older adults in Taitung City had higher demands with regard to this domain than the older adults in the other townships. Moreover, the scores demonstrated that due to differences in regional conditions, the demands for AFC varied considerably.

### 3.2. Service Providers: Analysis of Focus Group Interviews

The focus group interviews were conducted to understand the opinions of stakeholders from different areas regarding the current status of the AFC domains in Taitung County and its future policies. This study categorized Taitung County into four areas: downtown Taitung, the South-link region, the East Rift Valley region, and the East Coast region. The results regarding the stakeholders’ opinions on the eight domains of AFC are as follows:

For “outdoor spaces and buildings”, the service providers believed that public spaces and toilets lacked accessibility (downtown Taitung and the South-link region); that there were uneven roads and unclear signs (downtown Taitung); and that outdoor spaces required regular inspections and to be kept clean (the South-link region). The East Coast region was a special case because, for a long time, its public spaces did not require construction permits, resulting in a lack of reparations and maintenance. Even if funds were available, it is not possible to renovate this area. As for “transportation”, the service providers believed that due to its remote location, a severe lack of transportation caused inconvenient traffic situations, especially for aboriginal tribes in the East Rift Valley, the East Coast, and the South-link regions. The transportation and construction work in downtown Taitung did not consider the needs of older adults. Bus station signs were neither clearly placed nor easy to read (downtown Taitung and the East Coast region). Moreover, a serious shortage of low-floor buses was noted; older adults find it difficult to get on and off normal buses. Therefore, the number of low-floor buses should be increased in downtown Taitung and the East Rift Valley region. Furthermore, the participants noted that there were no bus shelters, and that weather shelters and chairs at bus stations were damaged. Aggravated by the inaccessibility of buses coupled with unfriendly bus drivers reduced the older adults’ willingness to take buses. Therefore, bus drivers should be trained to accommodate older adults in the South-link region.

For “housing”, the service providers stated that the amenities around residential areas were not adequate in downtown Taitung. Older adults relocate to live with their children in cities located in West Taiwan, leaving their houses untended in the East Coast region. Households and public facilities were in urgent need of improvement in the South-link region. The government’s residential subsidies for low-income households and aborigines should be extended to older adults, including those living in residential environments and facilities in tribes in the East Rift Valley and South-link regions. Overall, 95% of residences are without construction permits and are not eligible for subsidies for follow-up maintenance in the East Coast region. Finally, house and land prices are increasing in downtown Taitung. Both the vacancy rate of properties and the aging population is increasing in the townships along the eastern coastline; therefore, the government and households should take action in the East Coast region. As for “social participation”, the service providers suggested that older adults be invited to participate in planning decisions to better understand their demands (downtown Taitung). Older adults in remote areas are isolated and lonely. The government should organize activities in these areas, such as holding food events, to integrate the community. Older adults should be encouraged to engage in outdoor activities to expand their interpersonal relationships in downtown Taitung and the South-link region. Moreover, the extent of subsidies should be increased in the South-link region. If possible, older adults should be involved in productive activities that improve their self-esteem in the East Rift Valley region.

For “respect and social inclusion”, the service providers thought that most Aborigines were raised by grandparents and therefore needed to be cared for by others in the South-link region. Older adults’ nonlocal resources (such as relatives and children) are a pertinent resource. Social welfare groups should consider improving the connections between older adults and their children, which would reduce the fluctuation of social welfare groups in the East Rift Valley region. Young people’s understanding of life and ethics should be strengthened in the South-link region. Regarding “civic participation and employment”, the service providers thought that the resource inventory should be improved to better understand the strengths and interests of older adults to provide them with suitable paid work opportunities. Moreover, the service providers felt that employment opportunities and work ratios for older adults should be improved in downtown Taitung and the East Rift Valley region. However, the older adults living in tribes were born into peasant families and were not acquainted with modern society. Therefore, it is crucial that training is provided on modern life skills, for instance, offering courses about tribal life to pass on the traditional culture (the South-link region). As for unpaid jobs, older adults in the South-link region are unwilling to volunteer; therefore, volunteers in this region should be paid. Moreover, the Volunteer Service Act, which does not adequately cater for people in rural communities, should be appropriately amended (the East Coast region).

For “community and health services”, the service providers thought that rural areas in downtown Taitung, the South-link region, and the East Coast region lacked medical services. For the East-Rift Valley region, standards and regulations for establishing day care centers are too high. The education and services provided by local organizations in this region should be improved. Teachers, health care providers, and other experts should be invited to give community health-related information to older adults with different physical and psychological conditions in the South-link region. There is a severe shortage of health care providers in downtown Taitung. The number of people with dementia is becoming a serious problem, which has increased the burden of care and requires specialized care from social welfare units in downtown Taitung. The Taitung County government should pay for the health insurance premiums of older adults in downtown Taitung. Regarding “communication and information”, the service providers believed that government units were not well connected and those contact procedures should be simplified in the East Coast region. Institutions should enlarge the fonts on brochures so that older adults can read them. Moreover, they should make good use of information technology. However, because of an information gap in remote areas and older adults’ inability to use a computer, it is necessary that information is provided in brochures and through communication in understandable languages assisted by village offices and community-based associations in the South-link area region.

## 4. Discussion: Dialogue Analysis between Older Adults and Service Providers

This study compared the demands between older adults and service providers regarding AFC projects with regard to the satisfaction levels (rankings) of the older adults for the eight AFC domains. First, “respect and social inclusion” had the highest score; the older adults in the East Coast region were especially satisfied with this domain. The service providers did not propose any suggestions for improvement; they believed that most of the current activities offered older adults opportunities to develop friendships. Conversely, the South-link region was most in need of improvement in this domain, which was consistent with the service providers’ opinions. As per the questionnaire survey results, the older adults in this region thought that society, the media, and the government did not consider their needs. The service providers also stated that due to its remoteness, many older adults lived alone in tribes and raised their grandchildren there, which has led to a lack of proper care for both the older adults and the children. Therefore, to improve the older adults’ satisfaction level in this domain, the government should not only consider older adults’ needs, but also consider the role of older adults as caregivers. That is, activities should be designed to involve the participation of different generations, which reveals that this area requires more diversified assistance in terms of community-based labor and resources.

In terms of “community and health services”, the older adults in the East Rift Valley region had the highest satisfaction level. The service providers suggested that standards and regulations for day care centers should be relaxed and that local organizations be supported and improved to provide more comprehensive community education and services. The older adults in the South-link region had the lowest satisfaction level for this domain. Their views that community facilities have inadequate security and that there were insufficient public presentations on health issues were consistent with those of the service providers, who thought that there was not enough community education on health issues in remote areas.

Regarding “housing”, the older adults in the East Rift Valley region had the highest satisfaction level, especially regarding the space inside houses and the convenience of medical facilities, which was similar to the opinions expressed in the focus group interviews. However, older adults living in urban areas were the least satisfied with public or rental housing. The service providers in this area also thought that the medical facilities were too distant from households. Moreover, because of a recent increase in tourism in this area, land and house prices have risen to a point that the needs of older adults could not be satisfied.

Regarding “communication and information”, the older adults in the East Coast region had the highest satisfaction level, especially regarding clear instructions and special assistance for hospital services, which the older adults regarded as quick and convenient. The service providers emphasized that public sectors should simplify contact procedures because both parties (businesses and clients) expect to provide and receive service through clear and simple procedures. However, the older adults in the South-link region were the least satisfied with font sizes on brochures and signs in public spaces (such as signs for public toilets, directions, and billboards). The service providers also thought that due to an inadequacy of information available to tribes, the older adults’ inability to use a computer and the Internet, and large information gaps, it was necessary for village offices and community-based associations to communicate in print and in spoken language. Both the older adults and service providers agreed that public information should be readable for the older adults; otherwise, it would not achieve the purpose of providing necessary information.

Regarding “social participation”, the older adults in the East Rift Valley region had the highest satisfaction level. They said that social participation was convenient because they received notifications of activities from government agencies (government offices in cities, townships, and villages), which was consistent with the opinions expressed in the focus group interviews. The service providers in the area believed that the older adults should be involved in productive activities that improve their self-esteem. The older adults in Taitung City were the least satisfied with the costs of social participation, the level of participation from family members, the level of accessibility to activities for disabled people, and the diversity of activities. The service providers in this region believed that the construction industry, providers of assistive devices, and design departments should invite older adults to understand their needs to provide appropriate services.

Regarding “outdoor spaces and buildings”, the older adults in the East Coast region had the highest satisfaction level. According to the questionnaire results, the older adults were most satisfied with the community safety because life in this area is simple and security problems are not as serious as in the cities. But service providers have a different opinion, they stated that the transportation was inconvenient, especially in tribes because of their remote locations, and that a serious shortage of public transportation has resulted in the unclear placement of bus stations and unreadable signs, revealing that despite the level of safety in rural areas, transportation is still an urgent problem that requires improvement. The older adults in downtown Taitung were the least satisfied with the uneven roads, arcades, congested sidewalks, and road crossings, which was consistent with the service providers’ opinions. The service providers stated that the roads were uneven, signage was unclear, roads were unsuitable for older adults, sidewalks were not connected to specified green spaces, and that there were inadequate facilities for older adults and disabled residents.

Regarding “transportation”, the older adults in the East Coast region had the highest satisfaction level, especially regarding safety when taking a ride. But service providers thought that public transportation was insufficient in that region. The older adults in downtown Taitung were the least satisfied with the weather shelters, the number of seats at waiting areas at bus stations, amount of free transportation, convenience of getting on and off buses, and readability of signs at bus stations. These results were congruent with those of the service providers, which revealed that transportation and related infrastructure did not sufficiently consider the needs of disabled residents and older adults. More low-floor buses and readable bus information should be provided in this region.

Finally, all regions in Taitung County were dissatisfied with “civic participation and employment”. According to the views of the service providers, areas with a slightly better level of economic development (such as downtown Taitung and the East Rift Valley region) should assist older adults by developing their strengths to enable them to contribute to society. However, this strategy is not applicable to rural areas (such as the South-link and East Coast regions) because of their poor economic conditions. Older adults have considerable financial and health constraints; therefore, the purpose of working at their age is to earn an income to manage those constraints rather than to provide service or achieve personal goals. This also explains why the development of volunteering services is often particularly difficult in rural areas and within aboriginal tribes.

## 5. Conclusions

This study analyzed the current status of each AFC domain in Taitung County through questionnaire surveys and interviews with focus groups and proposed actions for future policies. The results and suggestions are as follows.

(1) Older adults were most satisfied with “respect and social inclusion” and were least satisfied with “civic participation and employment”. These domains require improvement accordingly.

When older adults are in different environments, what constituted an age-friend environment for them differed. For example, some older adults emphasized physical infrastructure and design; some emphasized the improvement of the social environment, including interpersonal relationships and social participation; and some desired changes in the government [1,5]. The results of this study showed that “Respect and social inclusion” had the highest average score among the eight AFC domains, which confirmed the influence of the concept of honoring the aged and respecting the wise in oriental cultures on older adults’ satisfaction. This phenomenon is particularly noticeable in Taiwan and Hong Kong [22], followed by “community and health services”, “housing”, “communication and information”, “social participation”, and “outdoor spaces and buildings”. “Transportation” and “civic participation and employment” had the lowest average scores, revealing that the Taitung County governmental offices in remote areas require a deeper understanding of these two domains.

This requires further planning and provision of services. For example, the frequency of public transportation was relatively low; moreover, there was a lack of bus stations in rural areas because of an insufficient number of passengers, causing inconvenience for the older adults when they needed to commute. Furthermore, “civic participation and employment” had a considerably low score (9.0), revealing that developing volunteering services in rural areas is difficult. Therefore, it is necessary to establish a more feasible system.

(2) Homogeneity existed between the older adults’ satisfaction levels in different regions and the service providers’ opinions. Those opinions are crucial for the planning of future policies.

Understanding how to construct inclusive and accessible urban environments through public opinions and participation is a key factor to promoting active aging and aging in place. It is the only way to determine the directions and visions for the future development of a city that are supported by the people [4]. The results showed that there was homogeneity between the older adults’ and service providers’ opinions regarding the eight AFC domains. The main reason for this is that the service providers either worked for institutions that provide services to older adults or were researchers in related fields and therefore had a thorough understanding of the older adults’ needs. Both parties shared similar opinions, such as inadequate transportation, employment, volunteer services, outdoor spaces and buildings, and social participation in Taitung County. Therefore, these domains should be included in future AFC policies in Taitung County to meet the needs and expectations of older adults.

(3) An in-depth analysis of older adults’ needs is recommended for understanding the discrepancies between their satisfaction levels and the level of services provided.

As previously mentioned, older adults in different environments had different needs for age-friendly environments [1]. This study revealed different results from older adults and service providers in different regions, showing that being in different regions, they had different needs with respect to age-friendly environments. Understanding how to integrate the differences between regions and developing effective policies are keys to the development of age-friendly cities.

Two phenomena are worth discussing when analyzing the results: first, the questionnaire results revealed that some regions where resources were scarce (such as the East Coast region) had higher satisfaction levels than regions with more resources. The dissatisfaction expressed by the older adults, however, did not necessarily mean that they lived in a poor condition. The results were dependent on the degree of their demands, which varied. For example, resources in the East Coast region were the most scarce, but this area had the highest satisfaction levels for “outdoor spaces and buildings”, “transportation”, “social participation”, “respect and social inclusion”, and “communication and information”. Another example is the perception that the infrastructure in downtown Taitung was more convenient than in the South-link, East Rift Valley, and East Coast regions.

However, the satisfaction level of the older adults in downtown Taitung for “transportation” was lower than that in those other areas, revealing that the older adults’ demands varied with their geographical conditions or personal statuses. In other words, older adults living in rural areas where resources are scarce are relatively easy to satisfy because their demands for quality of life and external conditions as well as for improvements are lower than those of the older adults in the other areas. This does not necessarily mean that there is no need for improvement. Therefore, it is suggested that various surveys be conducted to collect data about older adults, such as conducting field studies and in-depth interviews with older adults living in villages or within tribes, to understand their needs more specifically and to design appropriate policies accordingly.

(4) Economic development has a considerable influence on older adults in rural areas. Assisting the development of rural areas and the local industries of tribes might encourage young people to return to their hometowns and help with local AFC developments.

AFC-related studies have suggested that older adults should proactively participate in the development of AFC-related policies. Moreover, policy-makers should encourage and give older adults the right to develop age-friendly environments [1,2,5]. However, in reality, various factors, such as the demographics and economic conditions in a region, affected the social participation of older adults.

This study found that regional economic development affected the demands of the older adults. A lack of industrial development has caused a decrease in the number of young and middle-aged people, which is a serious issue in Taitung County. The lack of care that older adults receive from their families has led to grandparenting. If younger generations fail to provide economic assistance to their parents and grandparents, employment may become the focus of the older adults’ life. Consequently, social participation, activities related to health, and volunteer services are not feasible for older adults in regions with poor economic development. In other words, the poor economic conditions in rural areas that render older adults without family support and assistance from society hinder the promotion of AFC programs in those areas. Therefore, it is necessary to develop local industries and to gather resources through placemaking to promote employment and volunteering services, which would enhance the self-esteem of older adults and would involve them in their local environment.

(5) The needs of aboriginal older adults are easily neglected. It is suggested that tribal cultures be respected to enhance service providers’ sensitivity with regard to indigenous cultures.

Aborigines account for nearly one-third of the population in Taitung County. Therefore, issues concerning aboriginal older adults are worthy of attention. The lack of construction permits for public facilities has been a long-term problem in tribal areas, which needs to be solved. Public facilities should not be used and renovated without construction permits. Moreover, there is little public education about health in rural areas. Health education and medical services are not provided to aboriginal older adults in certain regions or tribes. This problem relates to the concept of “cultural care”; that is, overcoming the cultural barriers to improve their life would address cultural care with a health focus [23,24,25].

As previously mentioned, many of the tribal older adults lived alone, with some raising their grandchildren. Consequently, both the older adults and children were not cared for adequately and required community care and resources. Therefore, it is suggested that the cultural sensitivity of both service providers and politicians be enhanced to deeply understand older adults’ needs with an attitude of respecting and recognizing the cultural values of tribes. Implementing policies that accommodate a variety of needs and demands distinct from the Han Taiwanese values in Taitung County is challenging.

(6) There are different stages of development regarding the applicability of the AFC domains in Taitung County. Therefore, different townships should adopt different AFC strategies.

To summarize, considering the different stages of urban and rural developments is necessary when developing and promoting AFC projects in Taitung. This point is consistent with the arguments presented by Lehning et al., namely that the development of AFC policies should consider the aging population in cities and counties as well as the development of infrastructure, thereby revealing the importance of adopting diverse AFC policies. Moreover, aging is an ecological model when assuming that physical and social environments affect aging in place and quality of life during the aging process [7]. With regard to the four regions explored in this study, older adults in each region had different demands concerning the AFC domains, which was consistent with the service providers’ opinions. Therefore, local older adults’ demands regarding AFC projects should be considered first during urban development. Appropriate measures should be based on local conditions rather than adopting one measure and standard for all communities. Compared with Western countries, Taiwan’s aging population is growing rapidly. Local governments have already made some progress regarding AFC projects. However, understanding and managing the opinions of older adults and service providers in each county/city when promoting AFC projects according to their economic conditions is challenging. In other words, local governments should develop medium and long-term strategies and goals according to their conditions, resources, and problems. Even different regions in Taitung County, such as downtown Taitung and the East Rift Valley region, each require specific strategies for promoting AFC projects. Using Taitung County as an example, the short-term priorities for rural areas (such as the South-link and East Coast regions) should be to stabilize the lives of older adults. Without economic stability, older adults cannot participate in social activities. As for areas with better economic development (such as downtown Taitung and the East Rift Valley region), it is necessary to improve the opportunities for older adults to participate in environmental and social planning. Strategies should be adapted to local conditions, and they should be developed simultaneously to establish, gradually and effectively, a true age-friendly environment.

This study has several limitations. First, the participants were older adults participating in activities held by community care centers in each township, so they may not be a true representation of the older adult population in these areas. It is recommended that future studies adopt more comprehensive sampling methods to recruit older adults to ensure a representative sample (e.g., including older adults who do not participate in any activities held by those centers). Second, the quantitative questionnaire survey based on eight domains of AFC’s checklist and adjust question by situation of Taitung County. The quantitative questionnaire provides accurate methods to measure older adults’ satisfaction for eight domains of AFC, but this method does not directly reflect the needs of the older adults. It means that it is agreed that an older adults’ satisfaction questionnaire is considered to be a significant improvement tool for AFC. However, policy-makers and researchers still need to try more systematically and extensively methods to understand what older adults want, and provide a more age-friendly environment. Third, this study compared the views between older adults and service providers in four regions and emphasized the differences between them. How AFC policies should be applied based on the different development stages of older adults and service providers in different regions still awaits investigation.

## Figures and Tables

**Table 1:** Regional sample size and proportional allocation.

Regions	Number of People over 65 Years	Sampling Fraction	Expected Sample Size
Taitung City	12,403	2%	248
Beinan Township	2987	4%	119
Luye Township	1463	4%	59
Yanping Township	300	8%	24
Guanshan Township	1529	4%	61
Chihshang Township	2157	4%	86
Haiduan Township	246	8%	10
Taimali Township	1768	4%	71
Jinfeng Township	301	8%	24
Daren Township	367	8%	29
Dawu Township	782	8%	63
Donghe Township	1797	4%	72
Chenggong Township	2440	4%	98
Changbin Township	1690	4%	68
Lüdao Township	306	8%	24
Lanyu Township	311	8%	25
Total	30,611	3.56%	1090

**Table 2:** Data of the participants in the focus groups.

Category	Downtown Taitung	East Rift Valley Region	South-Link Region	East Coast Region
Local service providers (four persons)	Board directors of associations, social workers of day care centers, nursing supervisors of nursing homes, and nursing directors of home care institutes	Board directors of senior citizen clubs, executive secretaries of associations, social workers of day care centers, and coordinators of associations	Chairpersons of senior citizen clubs, personnel of associations, and board directors of associations	Board directors of senior citizen clubs, social workers of centers for Indigenous Family Services, supervisors of associations, and board directors of associations
Government Units (one or two persons)	Social workers from the Social Affairs, Department and section chiefs of the Health Bureau	Section chiefs of township offices and head nurses of public health centers	Officers of township offices and head nurses of public health centers	Head nurses of public health centers
Researchers and other experts (two persons)	University professors (public health and architecture)	University professors (public health and public affairs)	University professors (social work and architecture)	University professors (social work and public affairs)

**Table 3:** Reliability analysis of the eight domains of age-friendly city (AFC).

Order	Domains	Number of Items	Cronbach’s α
1	Outdoor spaces and buildings	7	0.8
2	Transportation	11	0.9
3	Housing	6	0.7
4	Social participation	9	0.9
5	Respect and social inclusion	8	0.9
6	Civic participation and employment	10	0.9
7	Communication and information	7	0.7
8	Community and health services	6	0.9

**Table 4:** Demographics of the interviewees.

Items	Categories	Number of People	Percentage (%)	Items	Categories		Number of People	Percentage (%)
Sex	Male	313	38.9	Partner status	With spouses/partners	Married	402	50.1
	Female	490	61.0			Cohabitate	14	1.7
Ethnicity	Aborigine	542	67.5		Without spouses/partners	Single	31	3.9
	Non-aborigine	261	32.5			Widowed	330	41.1
Age	55–64	153	19.0			Divorced	21	2.6
	65–74	366	45.6			Separated	5	0.6
	75–84	233	29.0	Place of residence	Taitung City		124	15.4
	85–94	48	6.0		Beinan Township		119	14.8
	Over 95	3	0.4		Luye Township		51	6.4
Education level	Illiterate	256	31.8		Yanping Township		20	2.5
	Literate	241	30.0		Guanshan Township		51	6.4
	Elementary school	205	25.5		Chihshang Township		85	10.6
	Junior high school	63	7.8		Haiduan Township		10	1.2
	Senior high school/vocational school	33	4.1		Taimali Township		58	7.2
	College/university	4	0.5		Jinfeng Township		15	1.9
	Graduate institute or higher	1	0.1		Daren Township		16	2.0
Living Pattern	Living alone	199	24.8		Dawu Township		24	3.0
	Living with spouses/partners	249	31.0		Donghe Township		56	7.0
	Living with children	187	23.3		Chenggong Township		76	9.5
	Three generations under one roof	122	15.2		Changbin Township		59	7.3
	Living with grandchildren	32	4.0		Lüdao Township		14	1.7
	Living with relatives	8	1.0		Lanyu Township		25	1.7
	Living in home care institutes	3	0.3					
	Others	3	0.4					
Receiving allowance	Yes	683	85.1	Volunteer experience	None		699	87.0
	No	120	14.9		Yes	<4 h/Month	41	5.1
Citizen status	Average households	757	94.3			4–8 h/Month	28	3.5
	Low- and middle-income households	18	2.2			>8 h/Month	19	2.4
	Low-income households	28	3.5		No opinion		13	1.6
					Declined to answer		3	0.4

**Table 5:** Average scores of the eight AFC domains.

Order	Domains	Number of Items	Total Scores	Mean ± Standard Deviation	Relative Scores	Ranking
1	Outdoor spaces and buildings	7	35	22.68 ± 3.82	64.8	6
2	Transportation	11	55	27.66 ± 15.89	50.3	7
3	Housing	6	30	20.33 ± 2.66	67.7	3
4	Social participation	9	45	29.44 ± 5.51	65.4	5
5	Respect and social inclusion	8	40	28.78 ± 5.16	72.0	1
6	Civic participation and employment	10	50	4.49 ± 9.86	9.0	8
7	Communication and information	7	35	23.60 ± 2.81	67.4	4
8	Community and health services	6	30	20.43 ± 3.3	68.1	2

**Table 6:** Scores of the townships regarding the eight AFC domains.

Regions	Townships	Outdoor Spaces and Buildings	Transportation	Housing	Social Participation	Respect and Social Inclusion	Civic Participation and Employment	Communication and Information	Community and Health Services
Downtown area	Taitung City	62.0	26.8	65.5	58.0	69.0	9.4	67.2	64.0
	Beinan Township	58.4	43.1	67.1	65.0	70.4	4.5	69.1	69.4
	Average	60.2	35.0	66.3	61.5	69.7	7.0	68.2	66.7
East Rift Valley area	Luye Township	67.0	59.7	69.0	65.1	61.9	2.2	72.7	67.9
	Yanping Township	69.3	76.0	76.7	74.2	74.4	14.0	67.4	72.2
	Guanshan Township	68.3	36.3	69.7	70.0	79.6	7.8	66.5	73.6
	Chihshang Township	65.7	56.6	69.7	68.6	76.0	22.1	62.6	70.8
	Haiduan Township	64.9	62.2	69.3	66.7	78.8	0.0	70.6	68.3
	Average	67.0	58.2	70.9	68.9	74.1	9.2	68.0	70.6
South-link area	Taimali Township	66.5	67.2	68.3	68.1	80.4	4.8	67.0	69.1
	Jinfeng Township	65.1	4.5	72.7	65.9	66.3	4.0	70.1	68.0
	Daren Township	63.4	37.5	60.8	63.3	62.2	48.0	63.4	62.5
	Dawu Township	64.4	59.2	67.1	63.7	62.5	3.5	63.2	66.1
	Average	64.9	42.1	67.2	65.3	67.9	15.1	65.9	66.4
East Coast area	Donghe Township	67.6	63.5	67.9	73.3	75.7	5.0	64.2	69.4
	Chenggong Township	66.3	66.3	67.5	66.2	77.8	3.6	69.5	71.0
	Changbin Township	71.2	71.1	70.9	67.2	71.6	0.0	73.7	67.7
	Average	68.4	67.0	68.8	68.9	75.0	2.9	69.1	69.4
Offshore islands	Lüdao Township	72.2	28.4	64.1	50.2	58.8	41.7	61.0	58.6
	Lanyu Township	58.2	36.0	58.0	58.5	61.7	20.6	63.2	57.2
	Average	65.2	32.2	61.1	54.4	60.3	31.2	62.1	57.9
